# Draft Genome Sequence of *Erwinia tracheiphila*, an Economically Important Bacterial Pathogen of Cucurbits

**DOI:** 10.1128/genomeA.00482-15

**Published:** 2015-06-04

**Authors:** Lori R. Shapiro, Erin D. Scully, Dana Roberts, Timothy J. Straub, Scott M. Geib, Jihye Park, Andrew G. Stephenson, Erika Salaau Rojas, Quin Liu, Gwyn Beattie, Mark Gleason, Consuelo M. De Moraes, Mark C. Mescher, Shelby G. Fleischer, Roberto Kolter, Naomi Pierce, Olga Zhaxybayeva

**Affiliations:** a Department of Organismic and Evolutionary Biology, Harvard University, Cambridge, Massachusetts, USA; b Forage and Bioenergy Research Unit, USDA-ARS, Grain, Lincoln, Nebraska, USA; c Department of Agronomy and Horticulture, University of Nebraska-Lincoln, Lincoln, Nebraska, USA; d Department of Entomology, The Pennsylvania State University, University Park, Pennsylvania, USA; e Department of Biological Sciences, Dartmouth College, Hanover, New Hampshire, USA; f U.S. Pacific Basin Agricultural Research Center, Tropical Crop and Commodity Protection Research Unit, USDA Agricultural Research Services, Hilo, Hawaii, USA; g Graduate Program in Bioinformatics and Genomics, The Pennsylvania State University, University Park, Pennsylvania, USA; h Department of Biology, The Pennsylvania State University, University Park, Pennsylvania, USA; i Department of Plant Pathology and Microbiology, Iowa State University, Ames, Iowa, USA; j Department of Environmental Systems Science, ETH Zürich, Zürich, Switzerland; k Department of Microbiology and Immunology, Harvard Medical School, Boston, Massachusetts, USA; l Department of Computer Science, Dartmouth College, Hanover, New Hampshire, USA

## Abstract

*Erwinia tracheiphila* is one of the most economically important pathogens of cucumbers, melons, squashes, pumpkins, and gourds in the northeastern and midwestern United States, yet its molecular pathology remains uninvestigated. Here, we report the first draft genome sequence of an *E. tracheiphila* strain isolated from an infected wild gourd (*Cucurbita pepo* subsp. *texana*) plant. The genome assembly consists of 7 contigs and includes a putative plasmid and at least 20 phage and prophage elements.

## GENOME ANNOUNCEMENT

*Erwinia tracheiphila*, the etiological agent of bacterial wilt disease of cucurbits, causes up to 80% yield losses in some varieties annually. The transmission of *E. tracheiphila* from infected to healthy plants requires an insect vector, namely, any of several species of neotropical luperine beetles (*Coleoptera*: *Chrysomelidae*). After infective beetles deposit frass containing *E. tracheiphila* onto floral nectaries or fresh leaf wounds, *E. tracheiphila* can enter the xylem, replicate, block the flow of xylem sap, and induce wilting symptoms. Death of the plant often occurs within weeks after the first onset of wilt symptoms. Here, we announce the draft genome sequence of an *E. tracheiphila* isolate obtained from an *E. tracheiphila*-infected wild gourd (*Cucurbita pepo* subsp. *texana*) from the Larson Experimental Station in Rock Springs, PA.

DNA was extracted from a single colony of *E. tracheiphila* culture grown in liquid nutrient broth (Difco), with a cetyltrimethylammonium bromide (CTAB)-based extraction protocol (1). Briefly, the cells were spun down and then lysed with 10% SDS, treated with proteinase K, RNase, and CTAB, precipitated in ethanol, and column purified (DNA Clean & Concentrator; Zymo Research, Irvine, CA). The SMRTbell template prep kit (Pacific Biosciences, Menlo Park, CA) was used according to the PacBio standard protocol “20-kb template preparation using BluePippin size-selection system,” including the DNA damage and end-repair steps and ligation to hairpin adapters. After DNA size selection of fragments >7 kb (BluePippin; Sae Science, Inc., Beverly, MA), the average library size was 27 kb. Three single-molecule real-time (SMRT) cells were run on a PacBio RS II instrument using a P4-C2 chemistry combination for an average 94× coverage. Adaptor trimming, quality filtering, and assembly were performed using the Hierarchical Genome Assembly Process pipeline (2). A starting seed length of 10 kb was used for the assembly, which resulted in 110,720 reads with a mean length of 6,560 bp and an *N*_50_ read length of 9,102 bp.

The high-quality draft assembly contains 7 contigs. One 49,303-bp contig contains plasmid conjugation genes and is likely a circular plasmid, and two contigs contain only phage genes and may be extrachromosomal phage genomes. The first phage contig contains 11,793 bp, with 54.5% G+C content and 17 coding sequences (CDSs). The second phage contig is 23,682 bp and has 54.5% G+C content with 43 CDSs, and the top-scoring BLASTp matches for most CDSs in the second phage contig are to a betaproteobacterial Mu-like phage. The remaining four contigs total 4,931,174 bp, with 50.6% G+C content and 5,414 CDSs predicted, with one of these contigs having 4,281,223 bp. Eighteen additional intact prophage regions were identified by PHAST (3). Prokka (4) was used as an *ab initio* gene predictor*.*

This genome sequence provides the first data point that can be used for functional characterization of this species. Whole-genome sequencing of additional strains isolated from different *Cucurbita* and *Cucumis* hosts will be important for investigating genetic diversity, the genetic basis of virulence, and host associations within the species.

### Nucleotide sequence accession numbers. 

This draft genome sequence has been deposited into NCBI under the accession no. JXNU00000000, BioProject PRJNA272881.
